# A vertex similarity-based framework to discover and rank orphan disease-related genes

**DOI:** 10.1186/1752-0509-6-S3-S8

**Published:** 2012-12-17

**Authors:** Cheng Zhu, Akash Kushwaha, Kenneth Berman, Anil G Jegga

**Affiliations:** 1 Department of Computer Science, University of Cincinnati, Cincinnati, Ohio 45229, USA; 2 Department of Pediatrics, University of Cincinnati, Cincinnati, Ohio 45229, USA; 3 Division of Biomedical Informatics, Cincinnati Children's Hospital Medical Center, Cincinnati, OH-45229, USA

## Abstract

**Background:**

A rare or orphan disease (OD) is any disease that affects a small percentage of the population. While opportunities now exist to accelerate progress toward understanding the basis for many more ODs, the prioritization of candidate genes is still a critical step for disease-gene identification. Several network-based frameworks have been developed to address this problem with varied results.

**Result:**

We have developed a novel vertex similarity (VS) based parameter-free prioritizing framework to identify and rank orphan disease candidate genes. We validate our approach by using 1598 known orphan disease-causing genes (ODGs) representing 172 orphan diseases (ODs). We compare our approach with a state-of-art parameter-based approach (PageRank with Priors or PRP) and with another parameter-free method (Interconnectedness or ICN). Our results show that VS-based approach outperforms ICN and is comparable to PRP. We further apply VS-based ranking to identify and rank potential novel candidate genes for several ODs.

**Conclusion:**

We demonstrate that VS-based parameter-free ranking approach can be successfully used for disease candidate gene prioritization and can complement other network-based methods for candidate disease gene ranking. Importantly, our VS-ranked top candidate genes for the ODs match the known literature, suggesting several novel causal relationships for further investigation.

## Background

In the USA, a rare or orphan disease (OD) is defined as a disease that affects fewer than 200,000 inhabitants [1]. According to an estimate, there are as many as 8000 ODs, many of which are known to be of genetic origin, affect children at a very early age and are life-threatening and/or chronically debilitating [2,3]. Although, the advent of next-generation sequencing technologies accelerates the disease gene discovery pipeline, the prioritization of candidate genes is still a critical step for disease-gene identification [4]. We [5], and several other earlier studies [6-9], have shown that genes associated with phenotypically close disorders tend to share molecular signatures which include similar expression profiles, participation in the same biological processes or pathways, protein interactions or complexes, literature co-citation. We have recently completed a global analysis of all ODs that have at least one known mutant gene associated (data from Orphanet [10] and the OMIM databases [11]) and show that the relationship between ODs cannot be fully captured by the gene-based network alone. Integrating diverse biomedical and genomic data types can facilitate hypotheses synthesis about disease causing mutant genes. Additionally, it can help in addressing an important question, namely, *are there any candidate genes related to known causal genes for a disease? *A useful way to approach this question is to rank the genes in a test set based on their similarity to a reference or 'seed' set. Such a "guilt by association" ranking approach has become an important way to prioritize candidate disease genes, such as the candidates found in genome-wide association or linkage studies [12]. The genes within a locus shown to be linked to a particular disease, for example, can be prioritized based on their similarities to a reference set of known genes for that disease. We and others have developed several computational approaches which perform this task automatically [4,13-23].

Network-based analyses have been equally successful in the identification and prioritization of disease candidate genes [6,7,24-31] especially where the genes are relatively less annotated. Network-based candidate gene ranking approaches can be broadly grouped into two categories: parameter-based and parameter-free methods. The parameter-based methods, such as PageRank with Priors (PRP [28]), Random Walk (RW [27]) and PRIoritizatioN and Complex Elucidation (PRINCE [7]), usually require additional auxiliary parameters that need to be trained by using available data sets. The PRP for instance needs a parameter β to control the probability of jumping back to the initial node [28], and the PRINCE algorithm uses a parameter to describe the relative importance of prior information [7]. Since selecting optimal parameters could be a challenge, parameter-free approaches are preferred and considered as more user-friendly [29]. Additionally, most parameter-based approaches take into account the global information in the entire network which often requires extensive computation. For example, in PRP, scores of all the nodes need to be updated iteratively until they converge. This process typically becomes extremely slow and inefficient especially when the network size is large. The parameter-free methods (e.g. Interconnectedness or ICN [29]), on the other hand, measure closeness of each candidate gene to known disease genes by taking into account direct link and the shared neighbors between two genes and tend to be less intensive computationally. The performance of parameter-free methods however is usually not comparable to parameter-based ranking approaches. Here, we report a novel network-based parameter-free framework for discovering and prioritizing candidate orphan disease genes. We specifically focus on two aspects: a) enhance prioritizing performance compared to current parameter-free methods and b) achieve a comparable performance to the parameter-based ones. We test, in a leave-one-out cross-validation setting, the utility of our approach in prioritizing genes for 172 ODs with at least five known causal genes (from Orphanet database [10]). We compare the performance of our method to two approaches, one each from parameter-based and parameter-free methods. To demonstrate the utility of our approach, we rank the immediate neighbors of known OD genes as potential novel candidate genes. The immediate neighboring gene sets were compiled using (a) protein interactions; (b) functional linkage network [32,33]; and (c) literature co-citations.

## Results and discussion

### Vertex similarity (VS) based candidate gene ranking

Hypothesizing that genes that are connected to one or more known disease genes ("seed genes") are also probably implicated in the same disease, our goal is to find such novel candidate genes with "strong" associations to the seed genes. Our proposed VS-based candidate gene ranking approach is based on guilt-by-association principle. Two nodes or vertices are considered similar if their immediate neighbors in the network are themselves similar (common biological process, pathway, etc.). This principle is used to build a self-consistent matrix formulation of functional similarity that can be evaluated iteratively using only knowledge of the adjacency matrix of the network (based on functional annotations of genes). To this effect, we consider similarity between two vertices (genes) as a measure of their association strength in a network. Thus, two vertices with a high similarity are likely to be strongly related. In order to find the similarities between the seed and the candidate or test set genes, we introduce a vertex similarity measurement in our algorithm. Vertex similarity which defines the similarity of two vertices based on the structure of network has been used for information retrieval in World Wide Web [34] and in social network analysis [35]. Similarity measurements, such as cosine similarity, have been successfully applied for computing similarity between documents which are described as vectors of keywords [36]. However, to the best of our knowledge, there have been no reports of using it as a measure to compute similarity between two genes in a functional network and use it for ranking candidate disease genes.

**Figure 1:**
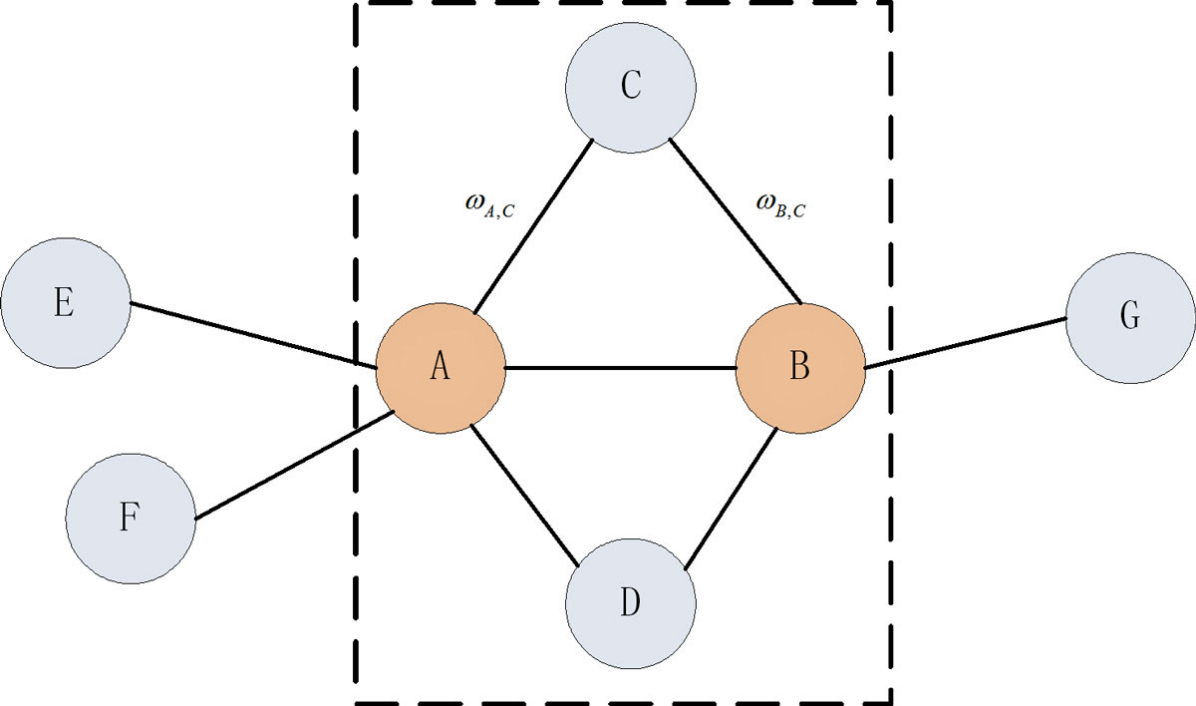
**Illustration of the connections between hypothetical genes A and B**. Each node represents a gene and each edge represents either a physical interaction or functional association. ω is the weight of each connection which in case of protein interactions is 1.

In our approach, when two genes are connected, the gene vectors are constructed based on protein interactions with the other neighboring genes. The shortest path is considered in cases where two genes are not directly connected. As illustrated in Figure 1, the similarity score *Sim(A, B) *between two genes A and B is defined as:

(1) $$Sim\left(A,B\right)=\frac{\overset{n}{\underset{i=1}{\sum }}\omega_{A,i}\times \omega_{B,i}}{\sqrt{\overset{n}{\underset{i=1}{\sum }}{\left(\omega_{A,i}\right)}^{2}}\times \sqrt{\overset{n}{\underset{i=1}{\sum }}{\left(\omega_{B,i}\right)}^{2}}}$$

where *ω_A, i _*represents the edge weight of node A to node *i*, and we define *ω_A, A _*= *ω*_*B, B *= 1 _(protein interactions); *n *is the number of the nodes which includes A, B and all the nodes that are directly associated with A and B. Equation 1 applies only when nodes A and B are connected and hence the value of n can be derived by

(2) $$n=\Gamma_{A}+\Gamma_{B}-\sigma_{shared}$$

where Γ*_A _*and Γ*_B _*represent the degree (number of connections or edges the node has to other nodes) of nodes A and B respectively, and *σ_shared _*= | Γ*_A _*∩ Γ*_B _*| and represents the number of shared neighbor nodes by both A and B.

When there is no direct connection between node A and node B, we try to find the shortest path between them. In this case, the similarity score will be derived by

(3) $$Sim^{*}\left(A,B\right)=\left\{\begin{array}{c} \prod_{k=1}^{K}Sim\left(C_{k},C_{k+1}\right)\\ 0 \end{array}\right)\begin{array}{c} if\begin{array}{cc} K\leq r & \end{array}\\ otherwise \end{array}$$

where *C_k _*is the node on the shortest path of A and B, and *r *is the discovery range that controls the maximum degree of separation (maximum *r *hops). In other words if the shortest path length between nodes A and B is more than *r *hops or if there is no shortest path between them, *Sim(A, B) *equals to 0.

The candidate genes in the test set are prioritized based on the similarity scores calculated from equation 1 and equation 3. For example, for a given disease *d*, each candidate gene is scored by summing up the similarity scores between the candidate gene and each of the seed genes from the training or seed set *S_d_*. The score of a candidate gene *i *is calculated as:

(4) $$score_{i}=\underset{j\in S_{d}}{\sum }Sim\left(i,j\right)$$

where *Sim(i, j) *is the connection score between gene *i *and *j*. All candidate genes are then ranked based on these scores.

### Comparison with other network-based prioritization algorithms

To compare the performance of our VS-based approach in candidate disease gene ranking, we select two methods, one each from parameter-based and parameter-free methods: PageRank with priors (PRP) [28] and Interconnectedness (ICN) [29]. Parts of implementation of PRP are done using JUNG (Java Universal Network/Graph; jung.sourceforge.net) framework [37] as described earlier [28]. To evaluate the performance of VS-based approach and compare it with two other methods, we used a leave-one-out cross-validation procedure. In each cross-validation trial, we removed a single OD causal gene ("target gene") from the data, and each of the 3 algorithms was evaluated by its success in assigning the rank to the "target gene" (see Methods for additional details).

**Figure 2:**
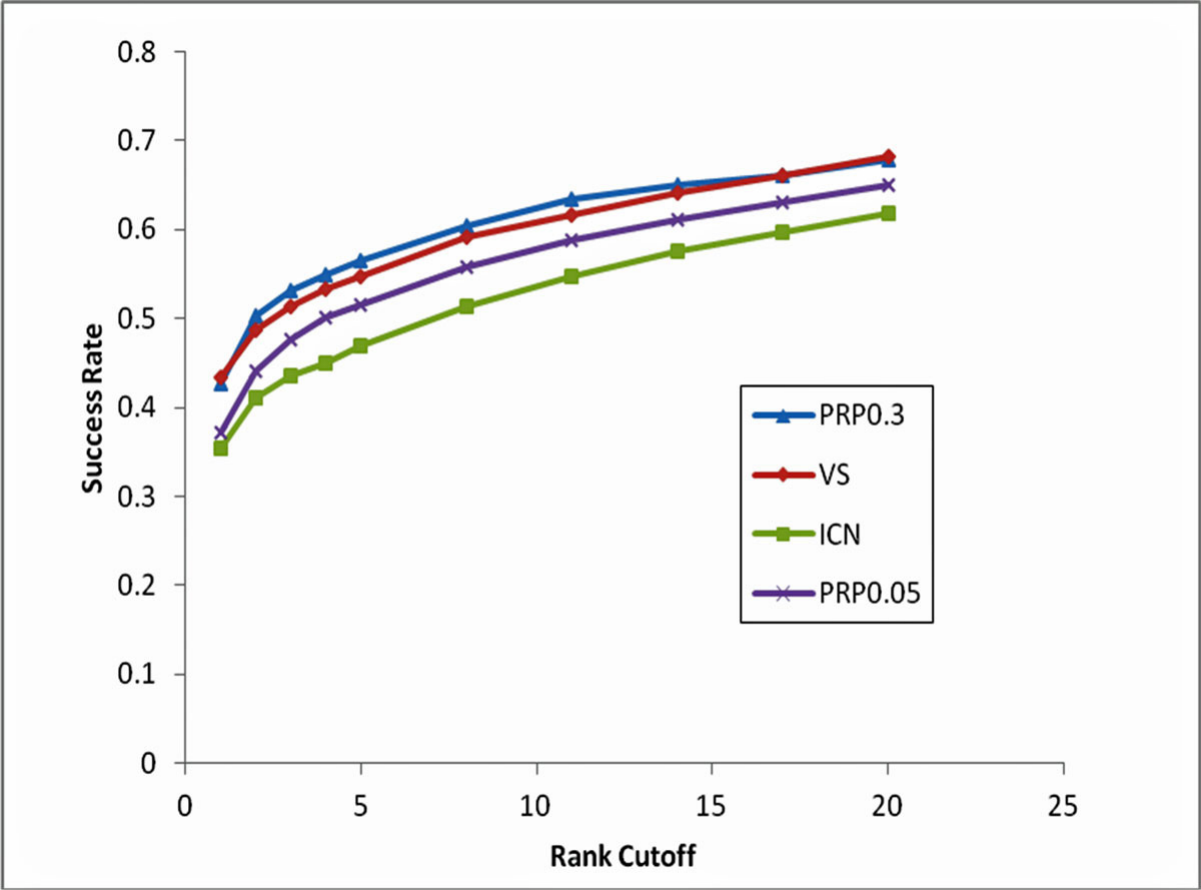
**Comparison of performance of parameter-based (PRP) and parameter-free (VS and ICN) methods in candidate gene ranking**.

We selected 172 ODs (ODs with 5 or more known causal genes) and 1598 OD causal genes for the cross-validation runs. Of the 1598 genes, we used 1312 which were in the protein interactome. The results from the leave-one-out cross-validation using the three approaches are presented in Figure 2. As can be seen from the Figure 2, when k (rank cut-off) = 1, both VS-based (parameter-free) and PRP (with back probability set to 0.3; parameter-based) methods, achieved the best performance with a success rate of ~43%. In other words, the target gene was top ranked in 568 out of 1312 cases (43.3%) using VS-based method. On the other hand with PRP (0.3 back probability), the target gene was top ranked 559 times out of a total 1312 cases (42.6%). ICN, another parameter-free method showed a lower success rate at 35.3% with the target gene ranked at top 463/1312 times. Expectedly, PRP (with back probability set to 0.05) showed a lower success rate than VS at 37.2% (488/1312) but was better than ICN.

When we increased the rank cut-off (k), VS-based approach performed equally well as PRP0.3. Additionally, compared to ICN, another parameter-free method, our VS-based approach performed better. We also note that VS outperformed PRP too when the back probability was set to 0.05.

**Figure 3:**
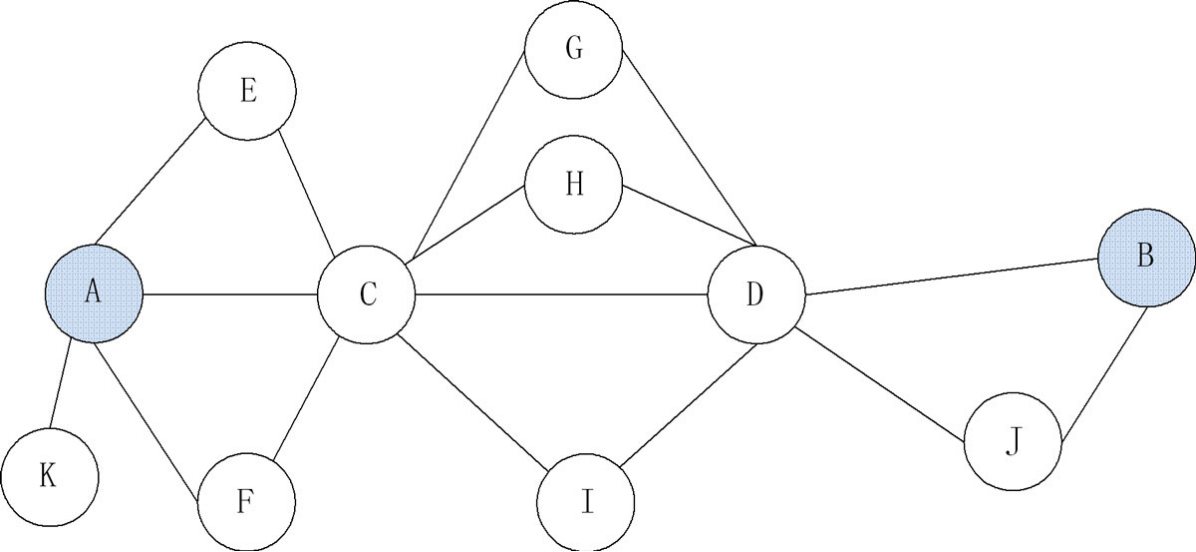
**An example network with indirect connections between hypothetical genes A and B**. Nodes A and B do not have a common neighbor and the shortest path connecting them is A-C-D-B.

The improved performance of VS over ICN we believe is because of the "extended guilt by association" [38] principle on which VS is based on. For example, if we consider a simple unweighted network (Figure 3; all edges equal to 1) where nodes A and B do not have a common neighbor and the shortest path connecting them is A-C-D-B. In this case, the ICN [29] score would be 0 because there is not even a single shared node between A and B. However, using VS, we can calculate the similarity between A and B (Sim(A, B) = Sim(A, C)*Sim(C, D)*Sim(D, B) = 0.276). Although, we have not performed an extensive analysis on disease gene connectivity, for the examples we have analyzed, we have found that several causal genes of a specific disease are connected indirectly (e.g., 3-step away).

However, since biological networks tend to have low diameters [39], we believe that low values of the steps/hops are preferable. Interestingly, a previous study provided examples of two real data applications where the number of hops or steps between disease causal genes (m) were set to two and reported that m = 2 was preferable over m = 1 [40]. Since the edge information between two genes may be noisy or incomplete, we believe that our VS-based approach for novel candidate disease gene ranking is desirable as it takes into account alternative measures of pairwise interconnectedness and is not just limited to direct interactions or having a shared neighbor node.

### Identifying and ranking novel OD candidate genes with VS-based approach

**Figure 4:**
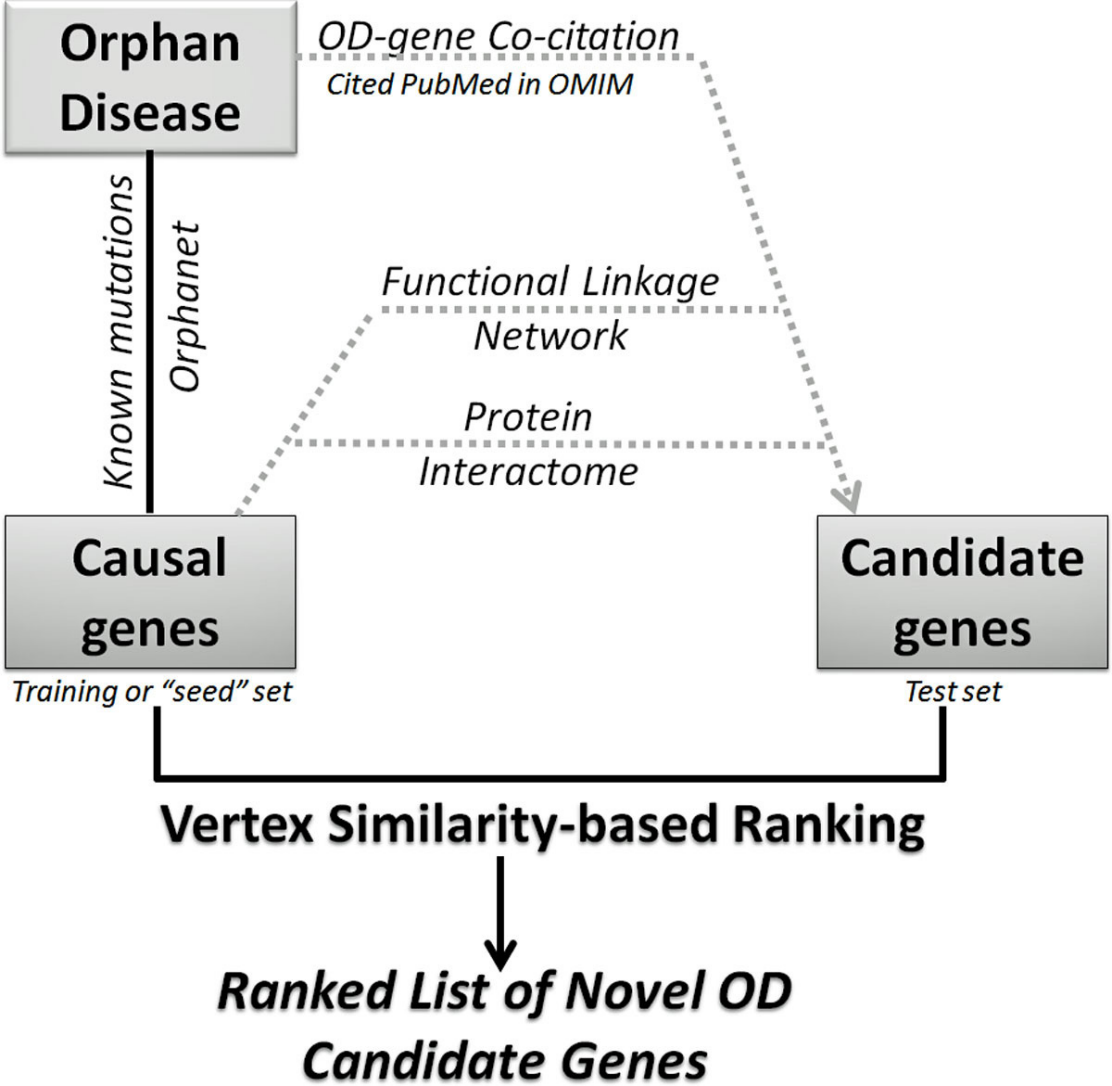
**Workflow for identifying and ranking novel OD candidate genes using VS**.

**Figure 5:**
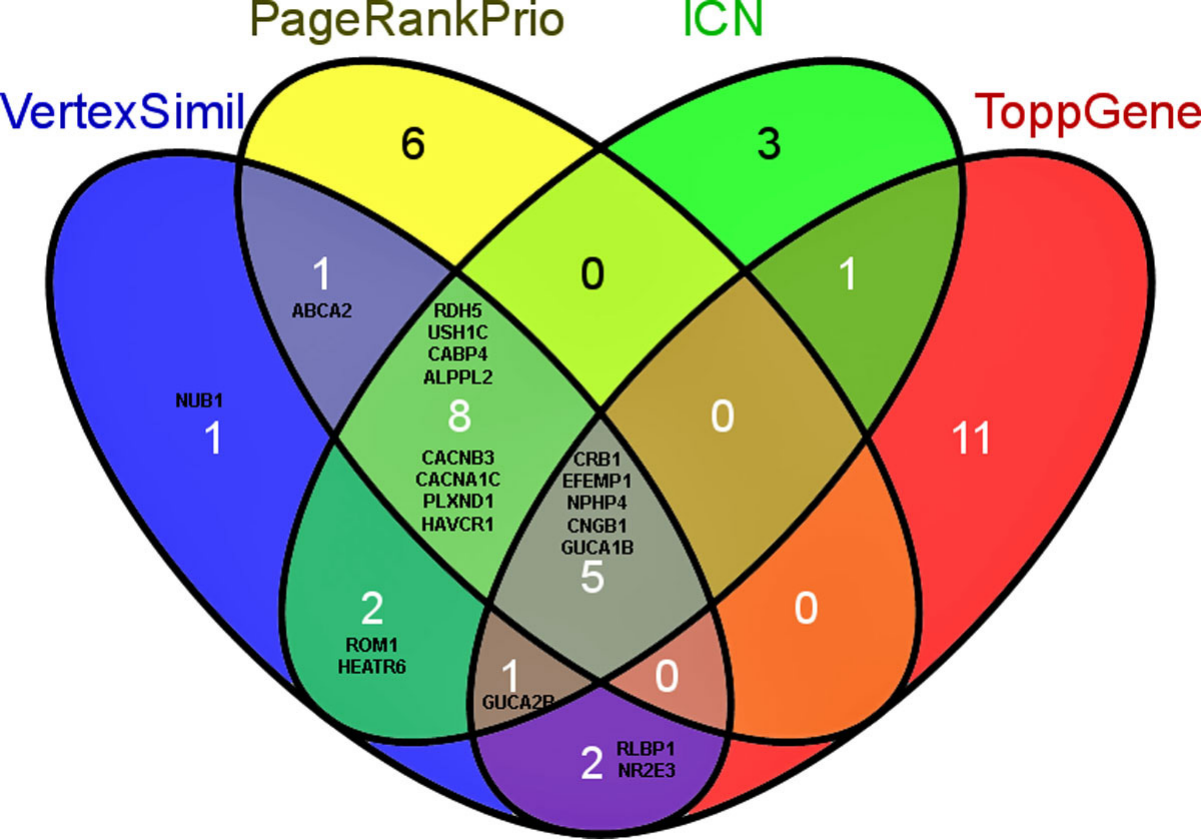
**Venn diagram comparing the top 20 ranked candidate genes for cone rod dystrophy using PRP (0.3), ICN, VS, and ToppGene**.

Having validated our method, we proceeded to execute our algorithm on several ODs with the goal of identifying and ranking potential novel candidate genes for ODs. We ranked candidate genes over the entire protein-protein interaction (PPI) network, and analyzed our top-five predictions for ten select ODs which have known protein interactions for all of their causal genes. The test set genes were compiled using several different sources comprising protein interactions, functional relatedness and literature co-citations. Briefly, for each causal gene of an OD, we extracted the immediate neighboring genes from the above mentioned resources (see Figure 4 and Methods for additional details). Table 1 shows the top-five predictions for ten ODs. We checked whether our VS top-ranked genes were already found to be associated with their query OD by searching online databases and scientific publications and found that most of the top candidate genes were already related to the respective OD. For example, the top five predictions for cone rod dystrophy are *CRB1, RDH5, USH1C, EFEMP1, CABP4*, which are genes associated with visual perception (Gene Ontology) and two of them (*CRB1 *and *CABP4*) are known to be involved in eye photoreceptor cell development and differentiation [41-43]. For this particular example, we also performed candidate gene ranking using PRP (with back probability set to 0.3) and ICN. We also used a functional annotation based candidate gene ranking method (ToppGene [16]) for ranking. When we compared the twenty top ranked genes from each of these three methods with VS-based ranking, there were five genes (*CRB1, EFEMP1, NPHP4, CNGB1 *and *GUCA1B*) common to all (Figure 5).

**Table 1:** Examples of orphan diseases and VS-ranked top 5 candidate genes

Orphan disease	No. of known causal genes	VS ranked top 5 candidate genes
Cone rod dystrophy	20	*CRB1, RDH5, USH1C, EFEMP1, CABP4*
Severe combined immunodeficiency	17	*CD3G, JAK1, ZAP70, IL2RB, IL4*
Fanconi anemia	15	*HES1, SAMD3, CYP19A1, XRCC3, USP1*
Zellweger syndrome	14	*PEX7, PHEX, ABCD2, ABCD1, ABCD3*
Autosomal dominant Charcot-Marie-Tooth disease, type 2	12	*STAT4, FAIM, MARCH5, STAT6, CRYGC*
Gonadal dysgenesis	12	*ZFY, ZFX, PTCH2, SOX9, AMH*
Hereditary nonpolyposis colon cancer	11	*MRC1, MSH3, CARKD, TRIT1, EXO1*
Papillary or follicular thyroid carcinoma	11	*CORO2A, ZBTB33, KIF11, AAAS, SEH1L*
Romano-Ward syndrome	11	*KCNE3, MINK1, KCNJ3, ALG10B, KCNJ9*
MODY syndrome	10	*GCKR, IDDM7, MAFA, ST6GAL1, INSRL*

Among other examples, *HES1*, the top ranked gene for Fanconi anemia is a novel interacting protein of the Fanconi anemia core complex and cells depleted of *HES1 *exhibit a Fanconi anemia-like phenotype [44]. The two top-ranked genes for gonadal dysgenesis, *ZFX *and *ZFY*, are known to function in sex differentiation and *Zfx *mutant mice are reported to have fewer germ cells than wild-type mice [45]. Likewise, maturity-onset diabetes of the young type (MODY syndrome) is linked to kinetic alterations and regulation of glucokinase activity [46,47] and in our ranking glucokinase receptor is the top ranked gene for MODY syndrome. Interestingly, a recent study in the Japanese families proposes GCKR as a susceptibility gene for familial diabetes [48]. While our ranking provides further support for the involvement of the top-ranked ranked genes in the investigated ODs, it also suggests that the top scoring candidates that are not previously associated with these ODs could be potential candidates for further research.

## Conclusion

The vertex similarity method (VS) is parameter-free approach for prioritizing candidate disease genes, where it calculates the similarity between nodes other than updating and training the parameters and data sets in every step. Through cross-validation experiments we show that VS outperforms ICN, another parameter-free method and that it is comparable to parameter-based methods such as PRP. We demonstrate the utility of VS-based parameter-free ranking approach in ranking OD candidate genes and importantly, these top ranked candidate genes for the ODs match the known literature, suggesting several novel causal relationships for further investigation.

Our approach however has some limitations. First, as with any training set dependent candidate gene ranking approaches, we assume that the OD causal genes we have yet to discover will be consistent with what is already known about an OD and/or its genetic basis which may not always be the case. Additionally, this also means that our approach currently cannot be used to rank novel candidate OD genes if an OD lacks known causal genes. Similarly, even if an OD has known causal genes but if there is no protein interactome data available then we cannot use VS for such cases. An alternative approach would be to consider other types of networks (coexpression or functional networks). Second, it is important to note that the prioritization by our approach can only be as accurate as the current protein interactome data are. Third, if a seed gene has only one known interaction then that interactant will be ranked higher.

## Methods

### Data resources

The ODs and causal gene information was downloaded from Orphanet [10]. We merged some of the OD subtypes of a single disease based on their given disorder names as described previously [5,8]. From this, we selected 172 ODs that have at least five causal genes. The total number of genes across 172 selected diseases was 1598. The human protein interactome used in this study was compiled from several resources [49-54] with both redundant interactions and self-loops removed.

### Prioritization methods

We performed a leave-one-out cross-validation using the 172 ODs and 1312 OD causing genes that exist in PPI network. We used the human protein interaction network as the global network to evaluate the prioritizing performance of VS and other two methods. The human protein interactome used in our study contains protein-protein interactions from large-scale yeast two-hybrid experiments [49,50], computational predictions [51], and curation of the literature [52-54], with both redundant interactions and self-loops removed. The assembled PPI network consists of 11,765 proteins and 69,167 interactions. During each set of a validation trial, one seed gene ("target gene") from one of the selected 172 ODs was picked out and mixed with 99 random genes from PPI network to form a test set of 100 candidate genes. The remaining seed genes of an OD were used as the training set. The test set genes were then prioritized using the three approaches: PRP (with back probabilities 0.3. and 0.05), ICN, and VS-based approach. During each run, the rank of the "target gene" was noted. We evaluated the performance of each algorithm in terms of the success rate versus rank cut-off (k). If the "target gene" is ranked among the top k in a particular validation run, it is considered as a 'success'. The validation runs are repeated until all the seed genes have been used as the target gene and their ranks are obtained. The "success rate" is defined as the ratio of successful validation runs and the total validation runs for all the existing OD genes from 172 ODs. The same strategy was followed for all the three algorithms. In case of PRP which is a parameter-based method, we selected a back probability of 0.3 since we have shown previously that the performance of PRP in ranking candidate disease genes was best at p = 0.3 [28].

### Test set genes for identifying and ranking OD candidate genes

For identifying and ranking novel OD candidate genes, we used the immediate neighbors of known OD genes as the test set. The immediate neighboring genes of selected ODs' causal genes were compiled based on (a) protein interactions; (b) functional linkage network [32,33]; and (c) literature co-citations. The protein interactome data as described earlier was compiled from several resources [49-54]. The functional linkage network-based candidate gene sets were derived from two resources: (i) HumanNet, a probabilistic functional gene network of Homo sapiens [33] and (ii) functional protein interaction network built upon expert-curated pathways [32]. The test set genes based on literature co-citations were compiled using the OMIM database. Briefly, for the selected ODs, we identified the corresponding OMIM records, which summarize results from publications about gene-disease relationships. For the OD mapped OMIM mapped records, we first extracted the cited literature (links to PubMed records for the references cited in an OMIM entry) in the OMIM records. Using this OD-related PubMed records, we extracted the related genes from the 'gene2pubmed' file from NCBI [55]. For a given OD with known causal genes, we pooled all neighboring genes (immediate neighbors or direct interactants) of causal genes from different sources and used it as a test set for ranking in the global protein interactome using VS-based approach.

## Competing interests

The authors declare that they have no competing interests.

## Authors' contributions

CZ, KB and AJ conceived the study design which was coordinated by AJ. CZ designed and implemented the VS-based candidate gene ranking approach and along with AJ and AK participated in the analysis and interpretation of results. CZ and AJ drafted the manuscript. All the authors have read and approved the final manuscript
